# Supplementary figures and images for: Eating breakfast and avoiding late-evening snacking sustains lipid oxidation
Source: PLoS Biol. 2020 Feb 27;18(2):e3000622. doi: 10.1371/journal.pbio.3000622 (PMC7046182; doi:10.1371/journal.pbio.3000622)

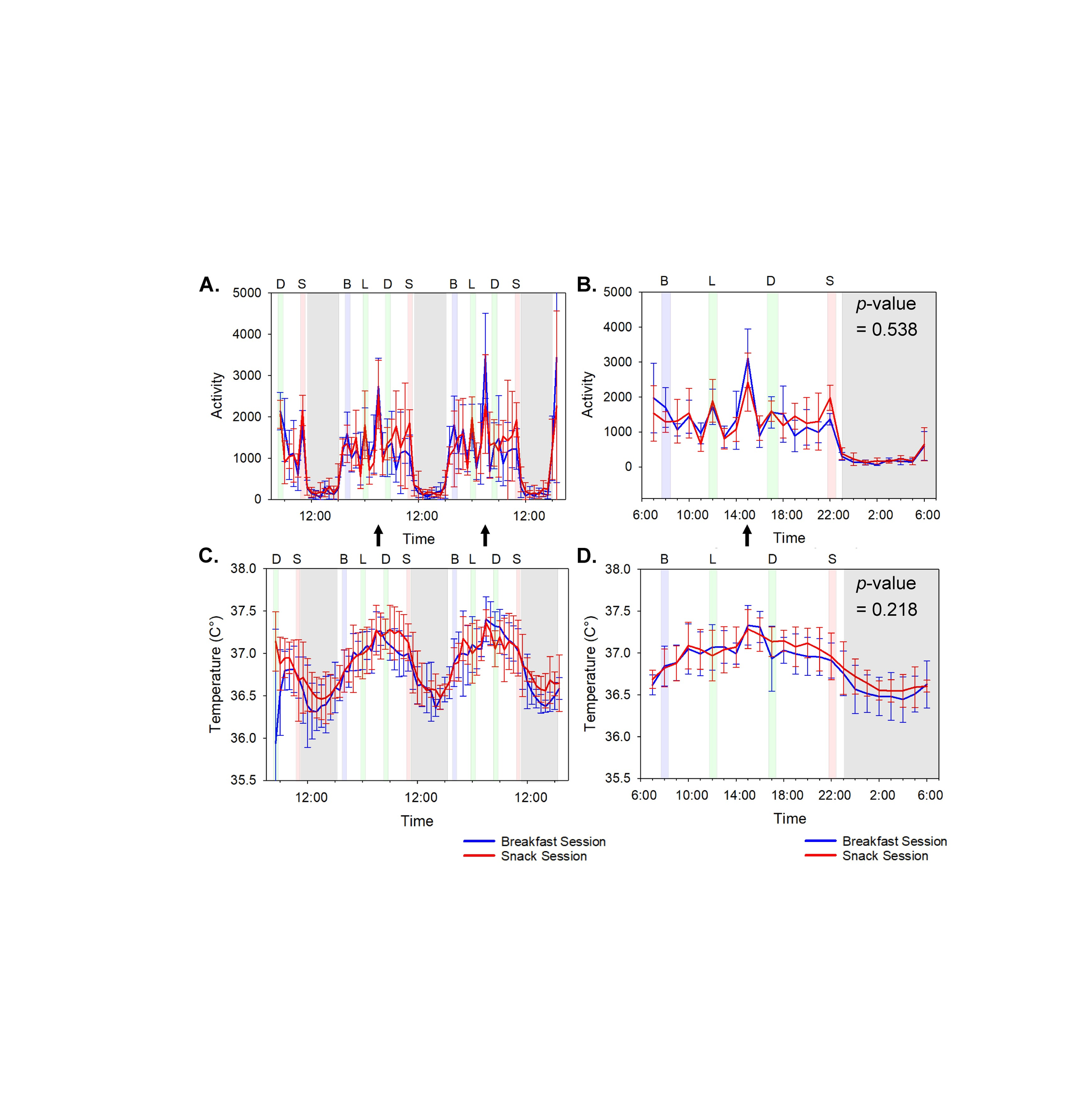

Supplement: S1 Fig — See S2B and S2C Table for underlying data. (A) Average wrist locomotor activity (measured in arbitrary units based on vector of magnitude) of all subjects for the 56-h time course. The blue line indicates values during the subjects’ Breakfast Sessions and the red line for the subjects’ Snack Sessions. Black arrows indicate the afternoon break during which subjects were allowed to exit the chamber for a 30-min break, during which the subjects were allowed a nonstrenuous walk. (This 30-min interval was excluded in other measurements because calorimetric readings were not being taken during this break.) (B) Average wrist activity of all subjects plotted modulo-24 h. Minute-by-minute activity data were averaged for all subjects into 1-h bins and aligned by clock time. The arrow denotes the 30-min break referred as noted in panel A. The p-value of 0.538 refers to a pairwise comparison of the average ([breakfast] − [snack]) difference values over the full 56-h time course for wrist activity. See S2B Table for the hour-by-hour statistical comparison of the breakfast versus the snack sessions. (C) Average CBT for all subjects over the 56-h time course. (D) Average CBT of all subjects plotted modulo-24 h. Minute-by-minute activity data were averaged for all subjects into 1-h bins and aligned by clock time. The p-value of 0.218 refers to a pairwise comparison of the average ([breakfast] − [snack]) difference values over the full 56-h time course for CBT. See S2C Table for the hour-by-hour statistical comparison of the breakfast versus the snack sessions. All panels: the blue line indicates values during the subjects’ Breakfast Sessions, and the red line the values for the subjects’ Snack Sessions. Shading indicates meals and lights off as in Fig 1B and 1C. Error bars indicate ± standard deviation (n = 6). CBT, core body temperature. (TIF) [file pbio.3000622.s001.tif]

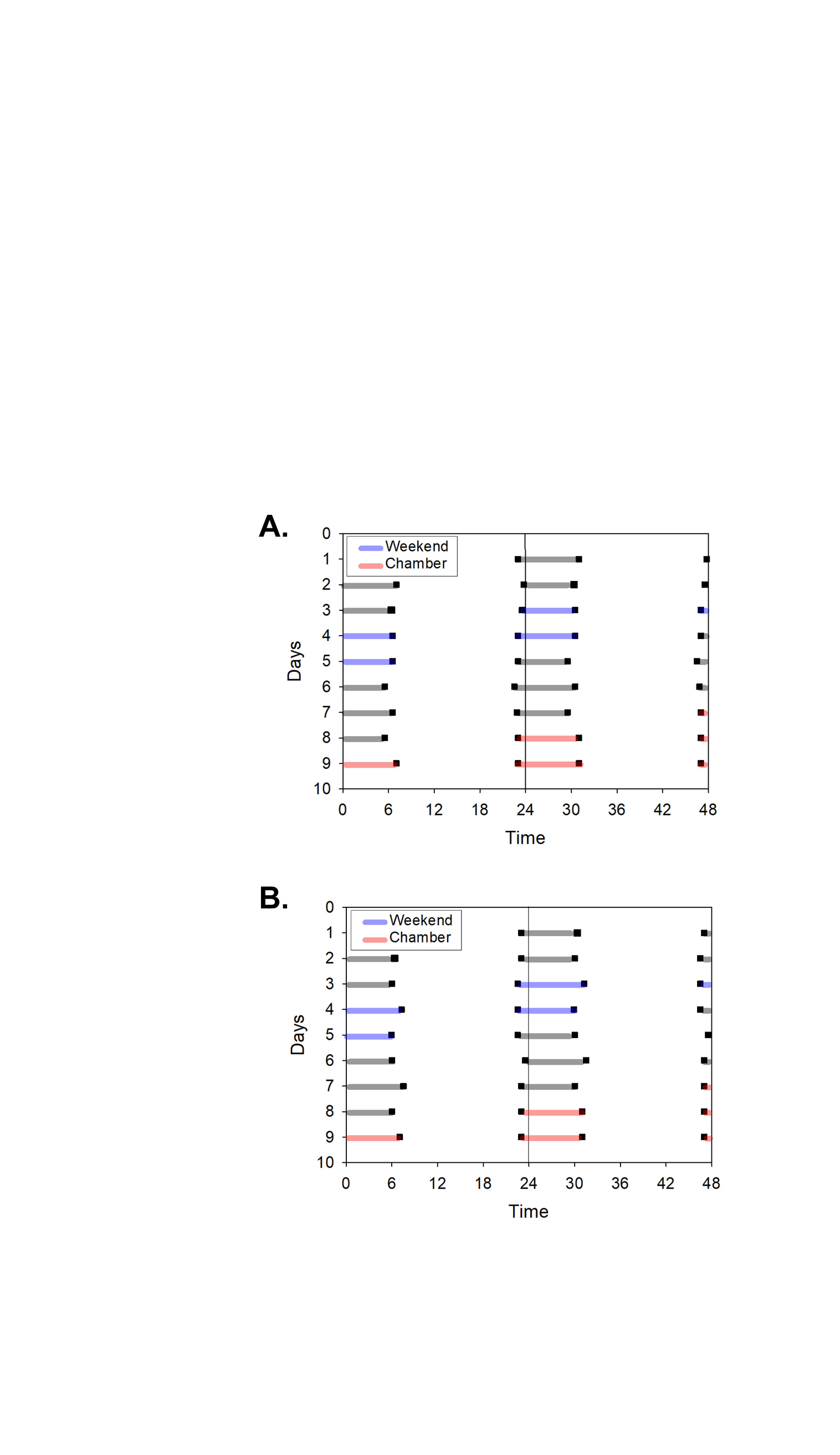

Supplement: S2 Fig — The subjects' self-reported bedtime and wake-up time for the week prior to entry into the metabolic chamber shows that the daily phasing of sleep was similar before and during the 56-h experimental time course. Black squares specify the time of bedtime and wake-up, with the horizontal lines indicating sleep episodes prior to entry into the metabolic chamber (blue horizontal lines) or during the 56-h experimental time course (red horizontal lines). Therefore, the subjects did not experience a phase shift of their daily cycle when they entered the experimental conditions in the metabolic chamber (compare with Table 1). (TIF) [file pbio.3000622.s002.tif]

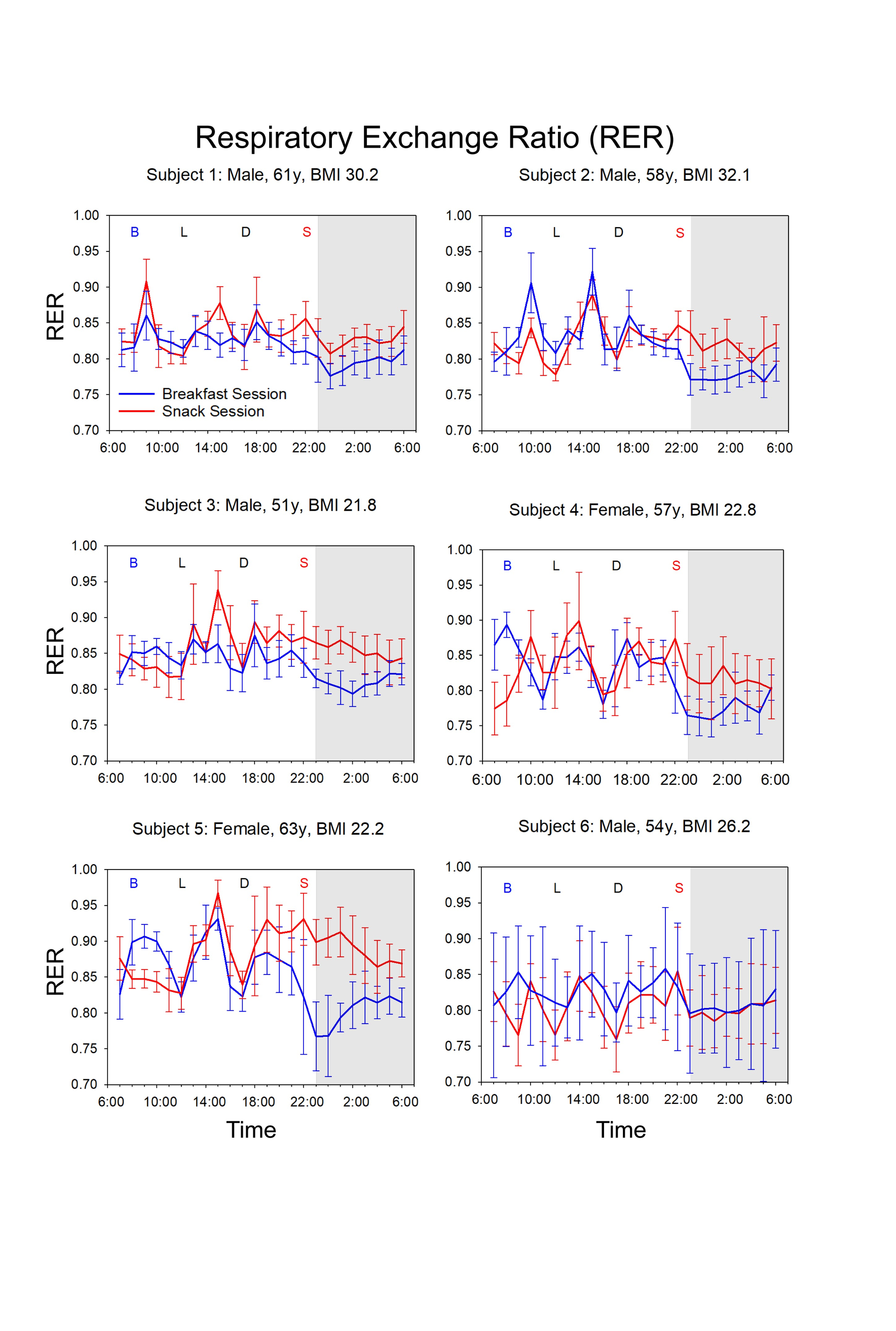

Supplement: S3 Fig — Average RER (VCO2/VO2) values for subjects 1–6 from their Breakfast Session (blue) and Snack Session (red) averaged into 1-h intervals. Error bars indicate standard deviation. RER, respiratory exchange ratio. (TIF) [file pbio.3000622.s003.tif]

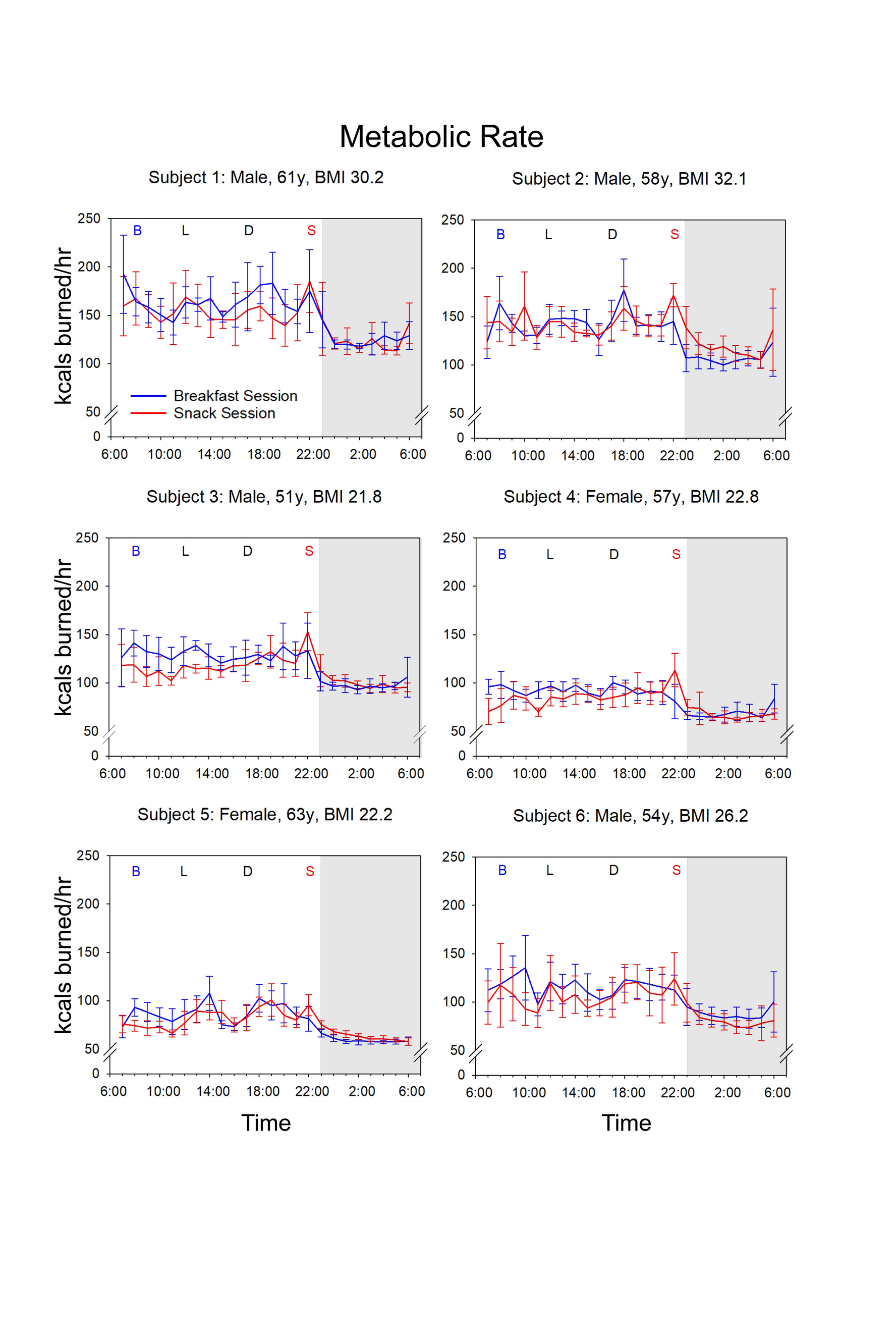

Supplement: S4 Fig — Hourly kcals burned for subjects 1–6 from their Breakfast Session (blue) and Snack Session (red) averaged into 1-h intervals. Error bars indicate standard deviation. MR, metabolic rate. (TIF) [file pbio.3000622.s004.tif]

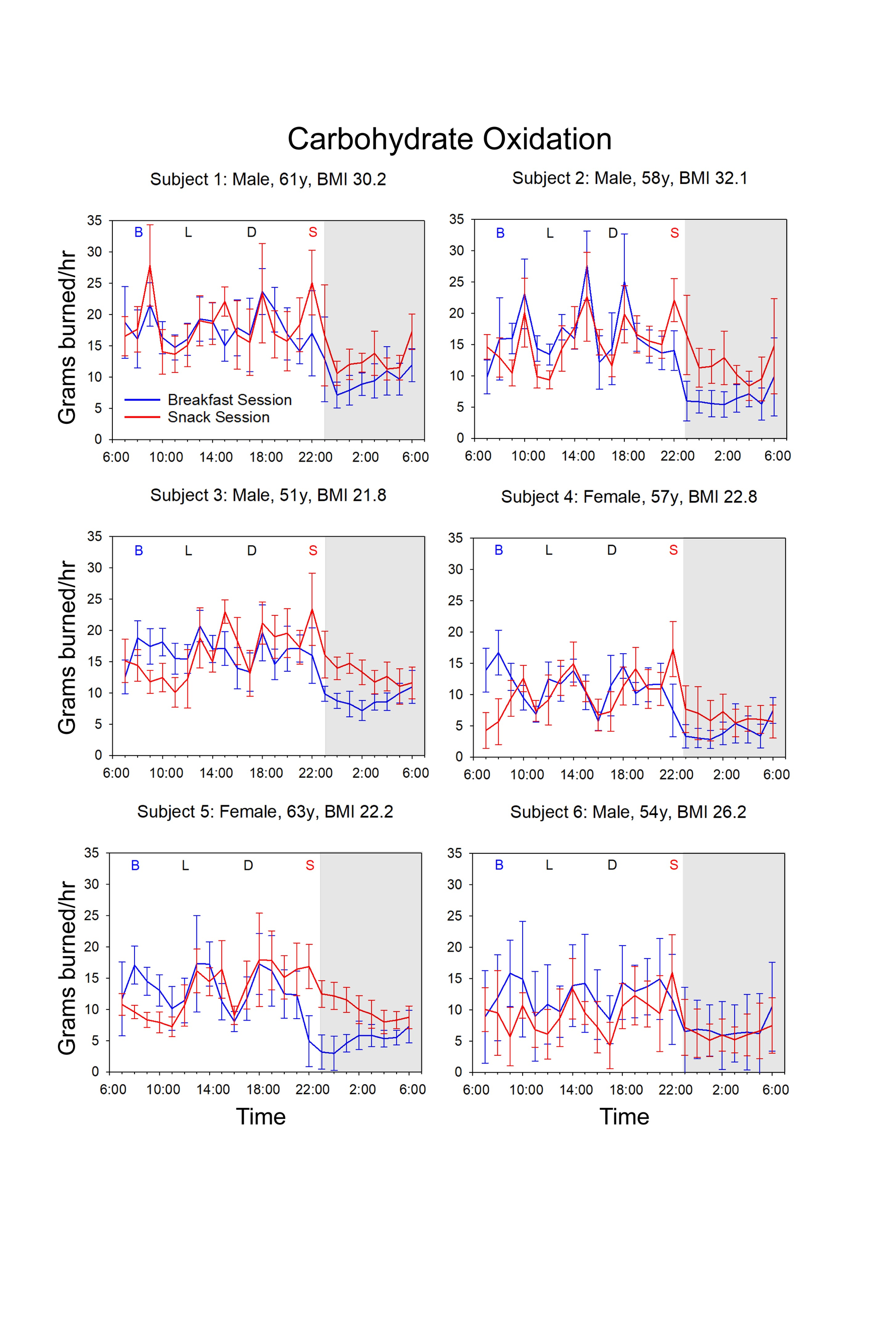

Supplement: S5 Fig — Hourly grams of carbohydrates burned for subjects 1–6 from their Breakfast Session (blue) and Snack Session (red) averaged into 1-h intervals. Daily CO values calculated from indirect calorimetry measurements as described [26,27]. Error bars indicate standard deviation. CO, carbohydrate oxidation. (TIF) [file pbio.3000622.s005.tif]

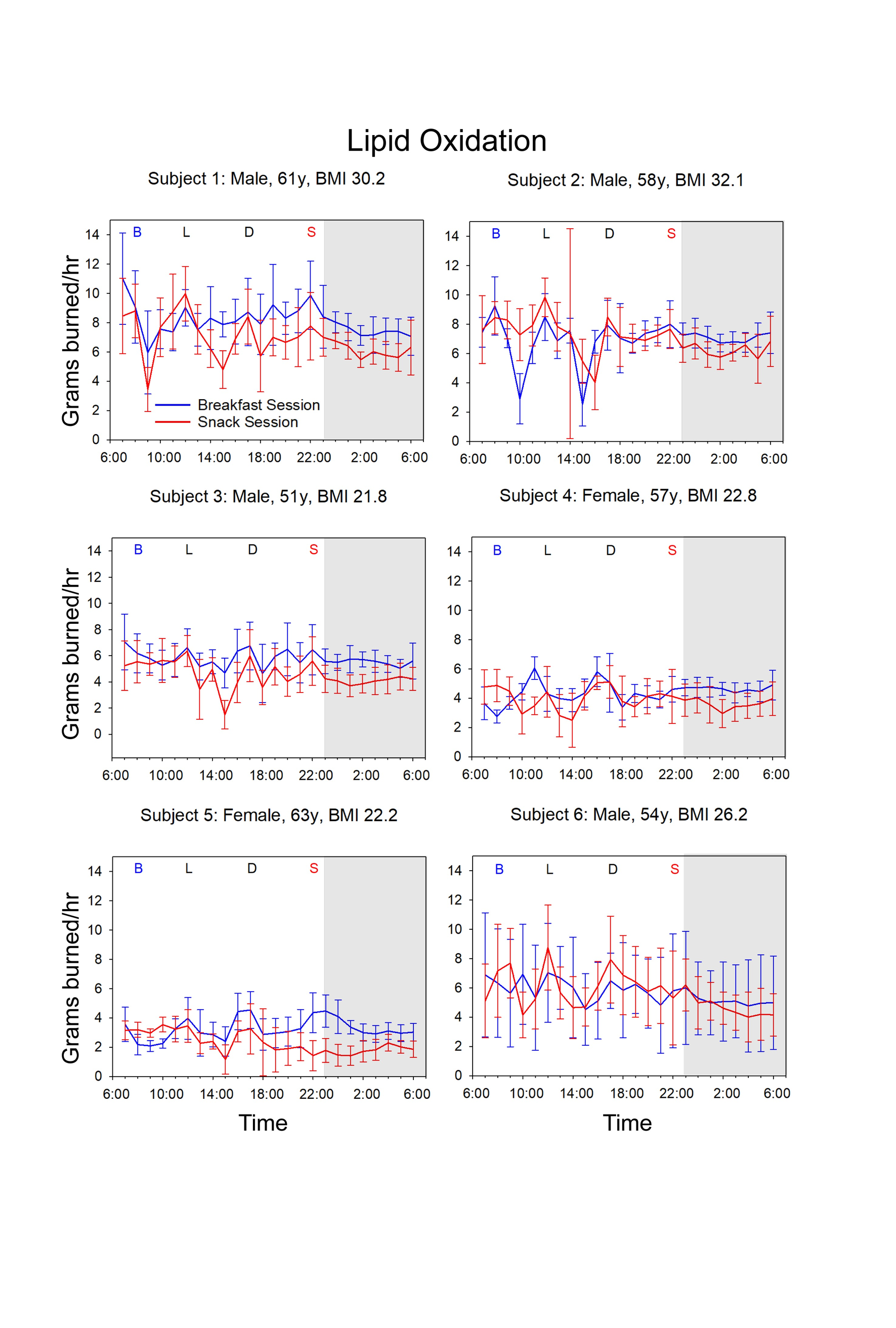

Supplement: S6 Fig — Hourly grams of lipids burned for subjects 1–6 from their Breakfast Session (blue) and Snack Session (red) averaged into 1-h intervals. Daily LO values calculated from indirect calorimetry measurements as described [26,27]. Error bars indicate standard deviation. LO, lipid oxidation. (TIF) [file pbio.3000622.s006.tif]

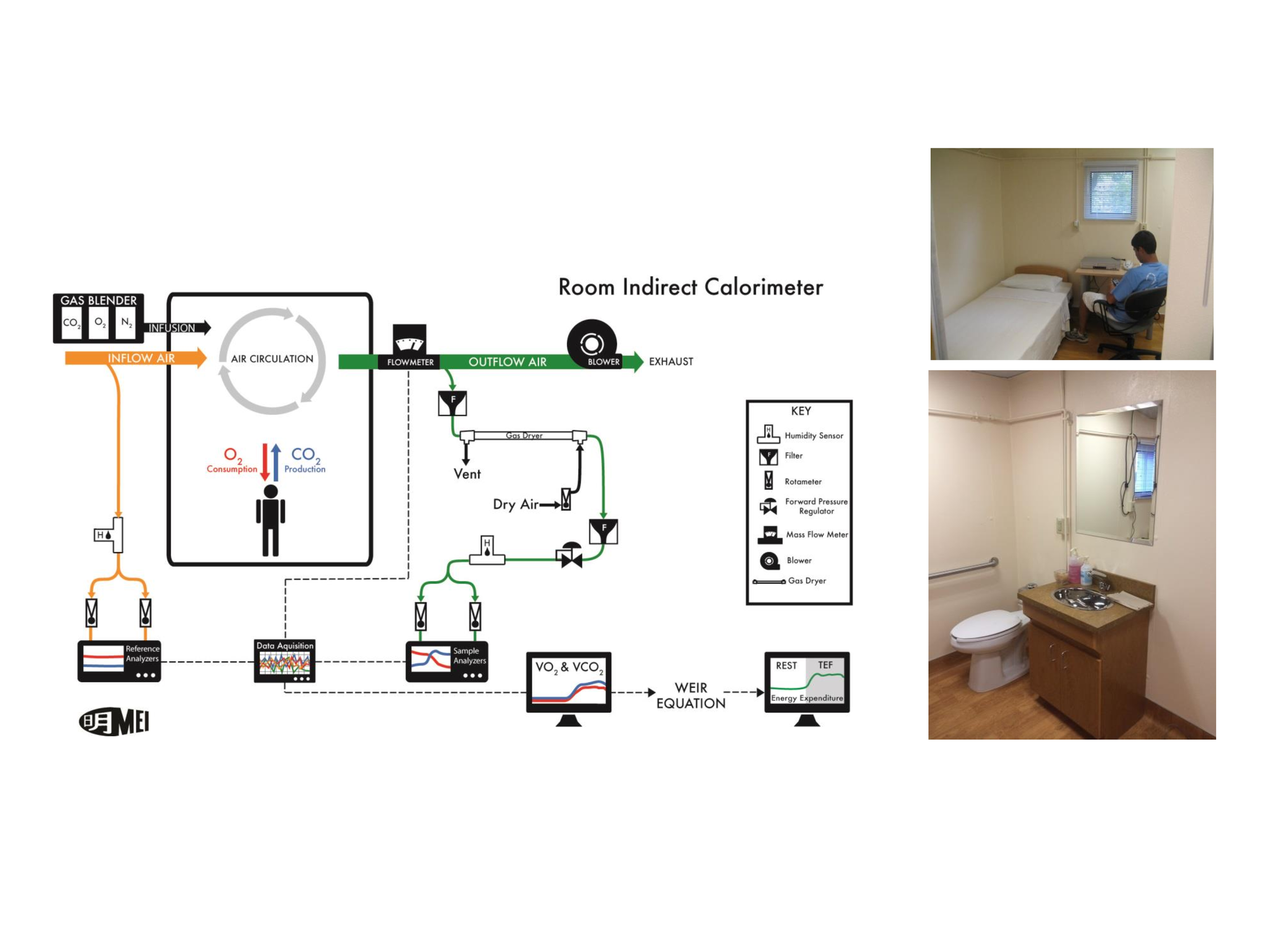

Supplement: S7 Fig — (TIF) [file pbio.3000622.s007.tif]
