# Supplementary material for: Eating breakfast and avoiding late-evening snacking sustains lipid oxidation
Source: PLoS Biol. 2020 Feb 27;18(2):e3000622. doi: 10.1371/journal.pbio.3000622 (PMC7046182; doi:10.1371/journal.pbio.3000622)
Supplement: S1 Text — (DOCX) [file pbio.3000622.s012.docx]

**S1 Text. Inclusion/Exclusion Criteria and Flow Diagram**

**Flow Diagram for Eligibility, Follow-up, and Analysis (CONSORT Flow Diagram)**

## Analysis

Analysed (n= 3)
♦ Excluded from analysis (n= 0)

Analysed (n= 3)
♦ Excluded from analysis (n= 0)

Allocated to Breakfast Session following washout period (n= 3)

Lost to follow-up (give reasons) (n= 0)

Discontinued intervention (give reasons) (n= 0)

Allocated to Snack Session following washout period (n= 3)

Lost to follow-up (give reasons) (n= 0)

Discontinued intervention (give reasons) (n= 0)

## Follow-Up

## Enrollment

Allocated to Breakfast Session first (n= 3)

♦ Received allocated intervention (n= 3)

♦ Did not receive allocated intervention (give reasons) (n= 0)

## Allocation

Allocated to Snack Session first (n= 3)

♦ Received allocated intervention (n= 3)

♦ Did not receive allocated intervention (give reasons) (n= 0)

Randomized (n= 6)

Excluded (n= 13)

♦  Not meeting inclusion criteria (n= 7)

♦  Declined to participate (n= 6)

♦  Other reasons (n= 0)

Assessed for eligibility (n= 19)

**Inclusion/Exclusion Criteria:**

Inclusion Criteria --subject must

- Be able to understand the study, provide written informed consent (in English), and be able to fill out the questionnaire
- Be male or female older than 18 years of age
- Have a normal BMI (20-25) or be obese (BMI more than 30)
- Have a normal basal glucose level (70-100 mg/dL)
- If female of childbearing potential, have a negative pregnancy test on study day

Exclusion Criteria-- subject must not

- Be pregnant or lactating
- Have known sleep, metabolic (e.g., diabetes), or gastro-intestinal disorders except obesity
- Had alcohol less than 24 hours before admission
- Require assistance with activities of daily living
- Have difficulty swallowing
- Be unable to complete a food and sleep diary
- Be smokers
