# Supplementary material for: Eating breakfast and avoiding late-evening snacking sustains lipid oxidation
Source: PLoS Biol. 2020 Feb 27;18(2):e3000622. doi: 10.1371/journal.pbio.3000622 (PMC7046182; doi:10.1371/journal.pbio.3000622)
Supplement: S2 Text — (DOCX) [file pbio.3000622.s013.docx]

**S2 Text. Subject Recruitment Questionnaire**

1. When do you sleep on a typical day?

a) Bedtime:­­­­­­­­­­­________________(e.g., 10:00-11:00 PM)

b) Wake-up time____________(e.g., 06:00-7:00 AM)

2. The following questions concern your typical meal times and food intake:

a) Do you normally eat breakfast? ___Yes ___No

If you answered Yes, what time do you usually eat breakfast? ____­­­­_______

Please give an example of what you might eat for a typical breakfast (e.g., piece of toast, bowl of cereal, or bacon, eggs, & toast, etc). ___________________________________________________

b) Do you normally eat lunch? ___Yes ___No

If you answered Yes, what time do you usually eat lunch? ______

Please give an example of what you might eat for a typical lunch ___________________________________________________

c) Do you normally eat dinner? ___Yes ___No

If you answered Yes, what time do you usually eat dinner? ______

Is dinner generally your largest meal of the day? ___Yes ___No

d) Do you frequently eat snacks between meals or after dinner? ___Yes ___No

If you answered Yes, what time(s) do you usually have your snacks relative to:

your main meals (check all that apply) ?

______In the morning between breakfast and lunch

______In the afternoon between lunch and dinner

______After dinner

e) How many caffeinated drinks do you drink daily? (Please answer in terms of the

number of cups of coffee or tea or number of portions of caffeinated soft drinks per

day): ­­­­­­­­­­­­­­­­________________________________________________

f) What is your average alcohol intake? (Please answer in terms of the number of

alcohol-containing drinks per week): ____________________________________

g) Do you have any dietary restrictions? ___Yes ___No

If you answered Yes, please describe what they are. ______________________

______________________________________________________________

3. Do you routinely exercise ___Yes ___No

If you answered Yes, please answer the following questions:

a) What type(s) of exercise do you do (e.g., jogging, swimming, yoga, etc.)

____________________________________________________

b) When (relative to your mealtimes) do you normally exercise?

____________________________

c) In a typical week, how many days do you exercise?

____1-2 ____3-4 ____5-7

4. Have you ever been diagnosed with any of the following diseases: ___Yes ___No

Esophageal stricture

Diverticulosis

Inflammatory bowel disease (IBD),

Peptic ulcer disease

Crohn's disease

Ulcerative colitis

5. Have you ever had gastrointestinal surgery: ___Yes ___No

6. The protocol for this study involves swallowing a capsule the size of a large vitamin. Do you have any difficulty swallowing: ___Yes ___No

7. Do you have any chronic medical problems not listed above (e.g., diabetes, high blood pressure, asthma, etc)

___Yes ___No

If you answered yes, please describe them._______________________

_________________________________________________________________

8. Are you currently taking any medications? ___Yes ___No

If you answered yes, please describe them. _______________________

_________________________________________________________________

9. Do you have any sleep disorders (e.g., sleep apnea, insomnia, sleep walking, restless leg syndrome, etc.)?

___Yes ___No

If you answered yes, please describe them. _______________________

_________________________________________________________________

10. Please complete the following demographic information:

Gender: _____male _____female

Age: _____ years

Ethnic group: _____ African-American _____ Hispanic

_____ Asian _____ Other

_____ Caucasian
