# Supplementary material for: Eating breakfast and avoiding late-evening snacking sustains lipid oxidation
Source: PLoS Biol. 2020 Feb 27;18(2):e3000622. doi: 10.1371/journal.pbio.3000622 (PMC7046182; doi:10.1371/journal.pbio.3000622)
Supplement: Attachment — Submitted filename: Response.pdf [file pbio.3000622.s016.pdf]

Vanderbilt University
Nashville, TN 37235 USA

Carl Johnson, Ph.D., Cornelius Vanderbilt Professor

Department of Biological Sciences
TEL: 615-322-2384; FAX: 615-936-0205
carl.h.johnson@vanderbilt.edu
January 10, 2020

Editors of *PLoS Biology*

Dear Madam/Sir:

We were very pleased by the enthusiasm expressed by the reviewers of our manuscript entitled "Eating breakfast and avoiding the evening snack sustains lipid oxidation." To convert our manuscript into a form suitable as a Short Report, we have moved one of our original text figures (old Fig. 2) to the Supplement and condensed two of our original text figures (old Figs. 4 & 5) to a single figure (now Fig. 3), thereby achieving a total of four text figures. In response to the reviewers' suggestions, we are honored to provide a revised version of the manuscript and a point-by-point response to the Reviewers. In the response below, the Reviewers' comments are in blue font and our responses are in black.

Reviewer #1:

Congratulations on a very interesting study! Overall, I think this paper by Kelly et al., provides a novel understanding of how the phase of eating alters metabolism, specifically lipid oxidation. The use of the metabolic chamber for 56 hours provides very robust data and as far as I am aware, has not yet been used to understand changes in eating phase in older, overweight adults. As the timing of eating is becoming an increasingly exciting field of study, many researchers and medical professionals will be interested in these findings as it provides novel insights into the importance of the phase of eating. I recommend that this paper is published with the minor revisions suggested below.

1. My largest concern is about the terminology used to describe the meals. First, breakfast is a very vague term and does not have a strong definition. For instance, NHANES surveys define breakfast as anything consumed before breakfast, during breakfast, or before lunch. Because of the subjective nature of the term breakfast, some readers (especially the media and general public) may take breakfast to mean very different things, including very early morning meals right as they wake up, which is not what was tested.
Secondly, the term 'snack' implies that it is a smaller meal. The authors do make it clear in the manuscript that they are the same number of calories, but for many that will not read the whole paper, this will likely be misleading, and should instead be referred to as a late-night meal.
Due to these likely misconceptions, I feel that changing the title and terminology throughout the text to refer to the different eating patterns as daytime and late-night eating, or early and late/delayed eating (as other papers have done – Hutchison et al., 2019) will more clearly explain the research. Additionally, the title is misleading as this paper does not fully determine if it is the presence of the breakfast, or the lack of night meal that leads to better lipid oxidation, but rather the phase of eating that made the difference. I agree the way that it is written is

technically correct, but I believe it will be misinterpreted as it can be avoided by changing the wording.

We appreciate the Reviewer's thoughtful comment here. We agree that the commonness of the terms for meals can lead to misinterpretations. Especially "snack" could mean a lot of different things to different people (as the Reviewer says, to many readers "snack" could mean a small snack of 100-200 kcals, while to many Americans a late-night snack might well exceed 1,000 kcal!). On the other hand, the immediate recognizability of the terms is also a benefit. Rather than using unfamiliar terms that could be cumbersome and confusing, we prefer to define clearly our terms in the beginning of the RESULTS, and continue to occasionally refer to the "snack" as a "late-night snack meal" to keep reminding the reader what we mean. We hope that this strategy is acceptable to the Reviewer.

Regarding the title, we'd prefer to use a relatively short and pithy title and let the Abstract speak definitively. Having said that, however, we would be amenable to any of these alternative titles and therefore if the Reviewer and/or the Editors prefer one of these alternative titles to our original title, we will be happy to substitute:

Eating breakfast and avoiding after-supper snacking sustains lipid oxidation

Eating breakfast and avoiding late-evening snacking sustains lipid oxidation

2. There are multiple papers that are relevant to this work that have not been cited and I would encourage adding to the introduction and/or discussion as the authors see fit.
• Early vs Delayed TRE:
o Hutchison, A.T., Regmi, P., Manoogian, E.N., Fleischer, J.G., Wittert, G.A., Panda, S. and Heilbronn, L.K., 2019. Time-Restricted Feeding Improves Glucose Tolerance in Men at Risk for Type 2 Diabetes: A Randomized Crossover Trial. Obesity, 27(5), pp.724-732.
• Timing of meals on metabolic health
o Jakubowicz, D., Wainstein, J., Landau, Z., Ahren, B., Barnea, M., Bar-Dayan, Y. and Froy, O., 2017. High-energy breakfast based on whey protein reduces body weight, postprandial glycemia and hba1c in type 2 diabetes. The Journal of nutritional biochemistry, 49, pp.1-7.
o Many other papers by Oren Froy's group
• Breakfast skipping and late-night eating are both associated with increased risk of coronary heart disease (good example of how late-night eating had a larger effect, but breakfast skipping was overemphasized as the cause of increased risk – need to make sure this does not happen here).
o Cahill, L.E., Chiuve, S.E., Mekary, R.A., Jensen, M.K., Flint, A.J., Hu, F.B. and Rimm, E.B., 2013. Prospective study of breakfast eating and incident coronary heart disease in a cohort of male US health professionals. Circulation, 128(4), pp.337-343.

Thank you for these specific suggestions. We certainly want to fairly cite our colleagues. However, our study is not specifically addressing health implications other than weight gain and metabolism, and therefore delving too deeply into coronary heart disease may be tangential. We have chosen to include the suggested Hutchison et al paper and a paper from the Froy group (about morning fasting) in the Introduction. We hope the Reviewer will consider these additions to suffice.

3. Please clarify in the methods if artificial sweeteners were allowed in the black decaf coffee.

Done!

4. In the text and figure 1 it shows that the snack was given at 10pm and lights off at 11pm, but in Supplementary Table 1 says snack is given at 11pm. Please correct or clarify.

Done!

5. Unlike the sleep data in the week leading up the metabolic chamber, the data on food intake leading up the metabolic chamber sessions is not provided in the supplement and is never mentioned except for the fact that they had them record it. Please provide a few sentences commenting on the eating pattern of the participants leading up to the trail. What was their eating window/when did they eat? How did the calories compare to the calories consumed while in the metabolic chamber? Etc.

We asked the subjects to record their sleeping and eating schedule prior to each visit to the chamber, and in the case of the sleeping schedules they carefully complied. However, in the case of the feeding schedules, the data were much sketchier. From the questionnaire, we knew their self-reported eating schedule in general and anecdotally the subjects said that they had kept to that schedule prior to the chamber visits. However, we do not have hard data for all the subjects on their feeding schedules specifically prior to entry to the chamber. Therefore, we have deleted the phrase in the M&M where we said that we collected their feeding habits for the one week prior to each chamber experiment.

6. Add units Fig 2A and B Activity – is it just A.U.? Also, for the comparison, was it total activity counts that was compared?

For the "old" Fig 2 (now S1 Fig), the activity was measured in arbitrary units based on vector of magnitude (that information is now included in the legend to S1 Fig). Also, the activity was total activity from the wrist actigraph (the wrist actigraph was used because the Winnebeck et al. [2018] determined that the wrist activity was most advantageous for assessing sleep quality).

7. States that sleep quality equivalent in both groups, but there are no stats of sleep efficiency provided. Please add and/or clarify how this was determined.

Based on the 2018 publication of Winnebeck et al., we assessed sleep quality as restlessness (measured by actigraphy) during sleep. That publication validates this particular measure, and we followed the methods described in that paper.

8. Be consistent about how figures are referenced in the text. Sometime figures are referred to by number and letter, sometimes only by number even when specifically referring to letters.

Done!

9. Some statements in the results seems more like discussion and makes assumptions about mechanism that were not directly tested, such as: "Apparently the late-evening snacking delays the clock induced switching between primarily carbohydrate-catabolic mode (higher RER values) and primarily lipid-catabolic mode (lower RER values." Because clock control was not directly tested, this should not be stated as a result. Please reword.

The writing style of the senior author (CHJ) is to include some interpretative material in the RESULTS because he believes that it allows the reader to more easily understand the flow

of reasoning from one experiment/analysis to the next. While he appreciates that some scientist writers prefer a strict delineation between RESULTS and DISCUSSION, we hope that the Reviewer will allow the senior author this particular eccentricity in the interests of his personal writing style.

In the specific case mentioned by the Reviewer, however, we agree that stating “clock-induced” in this sentence is not well supported, and therefore those words have been deleted.

Reviewer #2:

This is an excellent short report from Parsons Kelly and colleagues. Although preliminary, it fits well with the ethos of the short report article type. The data are presented well and completely, along with helpful supplementary data to allow further assessment of the raw data if necessary.

The principal conclusion of the report is that having an evening snack inhibits lipid oxidation (compared to having those calories at breakfast time), and in the long term, in metabolically "at risk" individuals, this could result in cumulative adipose tissue mass over time and the attendant effects on whole-body metabolism and notably obesity and diabetes.

The authors comment that RER is different between the two groups in the sleep phase, but not at other times. Other parameters (e.g. activity) are not different. However, I think that it would be interesting to analyze the data in a bit more depth than using a mixed model to detect differences between the groups. There doesn't appear to be any analysis of diurnal variation in the two groups. For example, comparing Fig. 1B and 1C, they look quite different over time, with the snack group (Fig. 1C) demonstrating a lower amplitude diurnal rhythm than the breakfast group (Fig. 1B). It would be instructive to estimate amplitude of these cycles (e.g. by using Circwave, JTK_Cycle, RAIN, or even PLS fitting). The authors should incorporate any arising points in their discussion. Other than this point, the analysis is very good, and the cross-over design is welcomed.

Those methods (Circwave, JTK_Cycle, etc.) are generally intended for high-dimensional data (e.g., thousands of genes) collected at relatively low temporal resolution. They also are designed to detect smooth daily variation (i.e., a rhythm with one peak and one trough per day). In our study, we have analyzed at high temporal resolution a small number of physiological parameters that are well known to exhibit daily variation but that do not always follow a smooth curve, and therefore we do not believe those methods are appropriate in this case. Our approach, which is specifically matched to our experimental design, allow us to quantify the overall differences between groups as well as the differences between groups at specific times of day.

However, inspired by the Reviewer’s comment, we have now used our mixed model approach to quantify–for each physiological parameter and each treatment group–the difference between the fitted maximum and the fitted minimum across the 24-h day, which is analogous to an amplitude. From this analysis, we found no significant changes in amplitude between breakfast and snack sessions in any of the parameters we measured (see table on following page). We do not believe that these results warrant inclusion in the main manuscript but we will include this analysis in the Supplement if the reviewer strongly urges.

Parameter	P-value from paired t-test
Activity	0.5542
RER	0.3592
Energy	0.9739
Lipid oxidation	0.436
Carbohydrate oxidation	0.2494
Temperature	0.4508

Reviewer #3:

This is an excellent and timely piece of research and clearly merits publication in a broad interest journal such as PLoS Biology.

Over the last ten years there has been enormous interest in the idea that, in addition to energy expenditure and what you eat, when you eat can have important effects on how food is metabolized and stored. There are a number of compelling mouse studies in this area, with correlative evidence from humans, but a clear interventional test of this phenomenon in humans under a well-controlled but pseudo real-world setting has been lacking. The authors have performed this study, using metabolic chambers and an elegant crossover design, in healthy middle-aged individuals; this being particularly pertinent as it reflects the demographic group most at risk from obesity in their country.

The data presented boils down to the analysis of a single experiment where oxygen consumption, carbon dioxide production, physical activity and core body temperature was measured with high temporal resolution over two sets of 3 days. Food intake was isocaloric between sessions, and the only difference was breakfast vs. bedtime snack, with the key finding being that bedtime snacks attenuated the nighttime dip in respiratory exchange ratio compared with the breakfast session, indicative of reduced lipid oxidation at night when feeding occurs later in the diurnal cycle. This data are convincing, with important implications for making achievable lifestyle interventions in obese individuals, and so important finding needs to be communicated to a wide audience.

Because this paper is likely to find a wide readership, there are some points of interpretation and presentation that I believe warrant some further consideration, as follows:

1) "The phasing or amplitude of the daily rhythms of master clock markers (plasma melatonin and cortisol), insulin, or plasma triglycerides is also not responsible, as reported by Wehrens and coworkers who found no differences in those rhythms in a meal timing study using a very similar protocol to ours [30]." I think this sentence is problematic for three reasons . . .the Reviewer inserts a thoughtful discussion of this issue here which I have deleted . . . . Alternatively, they may wish to simply wish to remove mention of insulin at this point in the manuscript. If I have misunderstood their argument, in which case please could they frame it more clearly.

We agree with the Reviewer here and will simply go with her/his alternative suggestion, namely to delete mention of insulin from this point in the manuscript. To make our essential point, it is really only the master clock markers (plasma melatonin and cortisol) that are

important for our interpretations here.

2) Statistics - the description of the linear mixed model, its parameters and implementation needs to be described in more detail. I assume that this more sophisticated analysis provides greater power, but it would be reassuring to the less-statistically literate to know whether a simple repeated-measures two-way ANOVA (regime vs time interaction) agrees with the linear mixed model. Moreover, it is good practice to report the power calculation that was performed before the experiment, which suggested that 6 individuals would be sufficient to detect a difference.

We have added the information regarding statistics requested by the Reviewer to the M&M. Additionally, please note that a repeated-measures two-way ANOVA is a special case of a mixed model. If the design is simple and there are no missing data, the results from these two approaches are similar. Since we had missing values for some time-points, a repeated-measures ANOVA would not use the entire data of that individual. However, a mixed-model would make better use of the whole dataset*. Thus, we applied the mixed model for our study.

*Krueger C,Tian L. A comparison of the general linear mixed model and repeated measures ANOVA using a dataset with multiple missing data points. Biol Res Nurs 2004;6:151–157.

Power calculation:
For an hourly paired t-test, a sample size of 6 has 80% power to detect an effect size of 1.435 (defined as mean difference/ standard deviation) with a 0.050 two-sided significance level. We applied a mixed model to perform an integrated analysis combining all hourly measurements together. The mixed model is more powerful than a traditional t-test comparison. The effective sample size of our study calculated from the mixed model is approximately 21**. We have added this information and reference to the M&M.

** C. Faes, G. Molenberghs, M. Aerts, et al. The effective sample size and an alternative small-sample degrees-of-freedom method. Am Stat, 63 (2009), pp. 389-399 (DOI: 10.1198/tast.2009.08196)

3) Analysis - by reporting the mean RER (e.g. Fig1B), the authors do not take into account any intra-individual variation in average RER. This is not a problem, but it seems plausible to me that one person might not have exactly the same basal RER are as somebody else due to genetic/epigenetic factors. Thus, if any variation exists, it does not seem unreasonable to me to correct for small differences in average RER (over the six days) between individuals as this would potentially remove a source of systematic error which is not relevant to their hypothesis, and thereby improve their sensitivity to detect differences that are specific to the two different feeding regimes.

RER is typically not normalized as it is a ratio of the volume of CO2 produced and exhaled by the subject over the volume of oxygen used (VCO2/VO2). It is likely that subjects of different body weights would produce different volumes of CO2/O2 due to varying energy requirements and thus normalization to body weight may seem reasonable. However, body weight normalization would not impact RER as it is based on a ratio of VCO2 and VO2 which would both be normalized. Furthermore, other research has suggested that normalizing to body weight leads to unintended bias (see Kailya & Schwartz 2011*) and as such normalization via body weight is generally not performed. Subjects were not genotyped in this study, however based on our exclusion criteria for subjects with preexisting metabolic disorders as well as our subject’s individual traces of RER, carbohydrate oxidation, and lipid oxidation (Figures S2,4,

and 5) we have no reason to believe there is any major difference in how lipids and carbohydrates are metabolized between our subjects.

*Kaiyala, K.J., & M.W. Schwartz. Toward a More Complete (and Less Controversial) Understanding of Energy Expenditure and Its Role in Obesity Pathogenesis. Diabetes Jan 2011, 60 (1) 17-23; DOI: 10.2337/db10-0909

4) Interpretation - dietary carbohydrates and lipids are used for many purposes besides respiration which are not explicitly considered. My understanding is that the major destinations of dietary carbohydrate are glycolysis+respiration (consumes O2, produces CO2) , glycolysis only (no O2/CO2 change), biosynthesis by pentose phosphate pathway (generates CO2, no O2 consumption), protein/lipid glycosylation (no O2/CO2 change), glycogen synthesis (no O2/CO2 change); whereas most dietary lipids are used for respiration via beta-oxidation (consumes O2, produces CO2), stored in lipid droplets (no O2/CO2 change), or used for membrane biosynthesis (no O2/CO2 change). RER would also be affected by any diurnal or feeding related changes in blood pH I imagine.

Thus, whilst it is entirely plausible that the changes in RER ratio over 24h, and between feeding regimes, mean exactly what the authors suggest they mean, there are several potential confounds to this interpretation which cannot be explicitly discounted without further experiments. Whilst this could be achieved by metabolic flux labelling with 13C-glucose, in no way would I expect the authors to perform any additional experiments since this would not affect the primary observation, that the night-time dip in RER is attenuated by a bedtime snack, only its interpretation. I would therefore suggest that some additional caveats to their preferred interpretation would be appropriate in the discussion section.

Indeed, their model (Fig6) does not really distinguish between the relative proportions of dietary vs stored carbohydrates/lipids that are being utilized as a function of time vs. feeding time - I would personally prefer that they propose a more detailed model that makes some explicit and testable predictions, which could be followed up in further rodent/human experiments, but the authors are free to disagree with my personal taste of course.

We agree with the reviewer that dietary carbohydrate and lipid have multiple fates some of which do not consume oxygen and/or produce carbon dioxide and potentially could be sensitive to feeding time. If you go back to the seminal studies where tracers were used to determine metabolic fates, in a net sense nearly all ingested lipid or carbohydrate that is not immediately oxidized is stored either as glycogen (primarily liver) or deposited as lipid (directly by esterification of dietary lipid or de novo lipid synthesis from dietary carbohydrate). Many metabolic pathways are used but if you follow the carbon to its final resting place in a 24 h period in a net sense it is either stored or oxidized. We agree that some of the carbon can be diverted to protein, DNA and RNA synthesis (and other pathways). This is readily detected as the Reviewer mentions by following the carbon using ^{13}C labeled substrates. But in a net sense it is a very small fraction. The dietary protein supplies the majority of the carbon for any newly synthesized protein. The total net number of cells (DNA and RNA content) in the body is constant, especially in the setting in this study where subjects are in energy balance.

Many pathways can be used to divert dietary carbon to their final fate. The pentose phosphate pathway (hexose monophosphate shunt) generates reducing equivalents (NADPH) for de novo lipid synthesis (no net CO_2 loss). For ribose synthesis, there is a release of CO_2 but in most cells that CO_2 can be “fixed” by other pathways such as synthesis of oxaloacetate from pyruvate (via pyruvate carboxylase) to serve as an anaplerotic substrate. There is a net glycolysis in muscle in the absorptive phase. Carbon not oxidized or stored as glycogen is

released as lactate/pyruvate or alanine and delivered to the liver for gluconeogenesis (Cori cycle). In the fed state, gluconeogenic carbon is diverted to glycogen deposition. So in a net sense, in this cycle carbon and oxygen is neither consumed nor produced. However, as energy is needed for gluconeogenesis, other substrates are oxidized to support the futile cycle. During the intervening time between meals, the glycogen (mostly liver glycogen) is released. From an energy balance point of view, ATP generated in muscle (glucose uptake or from muscle glycogen breakdown) glycolysis is offset by the ATP needed to resynthesize glucose in liver. In this cycle in net sense no energy was obtained from glucose oxidation. ATP however was used for this futile cycle.

There are a number of possible explanations as to how timing of meals affects net substrate oxidation. We hypothesize tissue glycogen pools (primarily liver), carbohydrate oxidation and de novo lipid synthesis are very sensitive to the feeding cycle. Indirect calorimetry measures net fat and net carbohydrate oxidation. Net carbohydrate oxidation is primarily the sum of the rates of glucose utilization for oxidation minus the rate of glucose used for de novo lipid synthesis. Net lipid oxidation is lipid oxidation minus de novo lipid synthesis [Frayn KN. Calculation of substrate oxidation rates in vivo from gaseous exchange. J.Appl.Physiol. 1983;55(2):628-34]. It is possible that the evening meal decreased net fat oxidation because de novo lipogenesis was increased. If both carbohydrate oxidation and de novo lipogenesis in the snack group were increased, they would offset one another and lead to no net change in carbohydrate oxidation. This could also limit the available carbon for hepatic glycogen deposition. But the higher de novo lipogenesis would decrease net fat oxidation. Future studies could probe how patterns of dietary intake alter de novo lipid synthesis, hepatic glycogen deposition and carbohydrate oxidation.

Thank you for your excellent suggestions and consideration !

Sincerely,

Carl Johnson
Cornelius Vanderbilt Chair in Biological Sciences
