# Supplementary material for: Eating breakfast and avoiding late-evening snacking sustains lipid oxidation
Source: PLoS Biol. 2020 Feb 27;18(2):e3000622. doi: 10.1371/journal.pbio.3000622 (PMC7046182; doi:10.1371/journal.pbio.3000622)
Supplement: Attachment — Submitted filename: Kelly et al, PloSBiol Review.docx [file pbio.3000622.s015.docx]

Overall, I think this paper by Kelly et al., provides a novel understanding of how the phase of eating alters metabolism, specifically lipid oxidation. The use of the metabolic chamber for 56 hours provides very robust data and as far as I am aware, has not yet been used to understand changes in eating phase in older, overweight adults. As the timing of eating is becoming an increasingly exciting field of study, many researchers and medical professionals will be interested in these findings as it provides novel insights into the importance of the phase of eating. I recommend that this paper is published with the minor revisions suggested below.

1. My largest concern is about the terminology used to describe the meals. First, breakfast is a very vague term and does not have a strong definition. For instance, NHANES surveys define breakfast as anything consumed before breakfast, during breakfast, or before lunch. Because of the subjective nature of the term breakfast, some readers (especially the media and general public) may take breakfast to mean very different things, including very early morning meals right as they wake up, which is not what was tested.

Secondly, the term ‘snack’ implies that it is a smaller meal. The authors do make it clear in the manuscript that they are the same number of calories, but for many that will not read the whole paper, this will likely be misleading, and should instead be referred to as a late-night meal.

Due to these likely misconceptions, I feel that changing the title and terminology throughout the text to refer to the different eating patterns as daytime and late-night eating, or early and late/delayed eating (as other papers have done – Hutchison et al., 2019) will more clearly explain the research. Additionally, the title is misleading as this paper does not fully determine if it is the presence of the breakfast, or the lack of night meal that leads to better lipid oxidation, but rather the phase of eating that made the difference. I agree the way that it is written is technically correct, but I believe it will be misinterpreted as it can be avoided by changing the wording.

2. There are multiple papers that are relevant to this work that have not been cited and I would encourage adding to the introduction and/or discussion as the authors see fit.

- Early vs Delayed TRE:
  - Hutchison, A.T., Regmi, P., Manoogian, E.N., Fleischer, J.G., Wittert, G.A., Panda, S. and Heilbronn, L.K., 2019. Time‐Restricted Feeding Improves Glucose Tolerance in Men at Risk for Type 2 Diabetes: A Randomized Crossover Trial. *Obesity*, *27*(5), pp.724-732.
- Timing of meals on metabolic health
  - Jakubowicz, D., Wainstein, J., Landau, Z., Ahren, B., Barnea, M., Bar-Dayan, Y. and Froy, O., 2017. High-energy breakfast based on whey protein reduces body weight, postprandial glycemia and hba1c in type 2 diabetes. *The Journal of nutritional biochemistry*, *49*, pp.1-7.
  - Many other papers by Oren Froy’s group
- Breakfast skipping and late-night eating are both associated with increased risk of coronary heart disease (good example of how late-night eating had a larger effect, but breakfast skipping was overemphasized as the cause of increased risk – need to make sure this does not happen here).
  - Cahill, L.E., Chiuve, S.E., Mekary, R.A., Jensen, M.K., Flint, A.J., Hu, F.B. and Rimm, E.B., 2013. Prospective study of breakfast eating and incident coronary heart disease in a cohort of male US health professionals. *Circulation*, *128*(4), pp.337-343.

3. Please clarify in the methods if artificial sweeteners were allowed in the black decaf coffee.

4. In the text and figure 1 it shows that the snack was given at 10pm and lights off at 11pm, but in Supplementary Table 1 says snack is given at 11pm. Please correct or clarify.

5. Unlike the sleep data in the week leading up the metabolic chamber, the data on food intake leading up the metabolic chamber sessions is not provided in the supplement and is never mentioned except for the fact that they had them record it. Please provide a few sentences commenting on the eating pattern of the participants leading up to the trail. What was their eating window/when did they eat? How did the calories compare to the calories consumed while in the metabolic chamber? Etc.

6. Add units Fig 2A and B Activity – is it just A.U.? Also, for the comparison, was it total activity counts that was compared?

7. States that sleep quality equivalent in both groups, but there are no stats of sleep efficiency provided. Please add and/or clarify how this was determined.

8. Be consistent about how figures are referenced in the text. Sometime figures are referred to by number and letter, sometimes only by number even when specifically referring to letters.

9. Some statements in the results seems more like discussion and makes assumptions about mechanism that were not directly tested, such as: “Apparently the late-evening snacking delays the clock induced switching between primarily carbohydrate-catabolic mode (higher RER values) and primarily lipid-catabolic mode (lower RER values.” Because clock control was not directly tested, this should not be stated as a result. Please reword.
